# Analysis of personality traits and their influence on the quality of life of postmenopausal women with regard to genetic factors

**DOI:** 10.1186/s12991-016-0110-6

**Published:** 2016-09-08

**Authors:** Daria Schneider-Matyka, Anna Jurczak, Agnieszka Samochowiec, Beata Karakiewicz, Małgorzata Szkup, Anna Grzywacz, Elżbieta Grochans

**Affiliations:** 1 Department of Nursing, Pomeranian Medical University in Szczecin, ul. Żołnierska 48, 71-210 Szczecin, Poland; 2 Department of Clinical Psychology, Institute of Psychology, University of Szczecin, Szczecin, Poland; 3 Public Health Department, Pomeranian Medical University in Szczecin, Szczecin, Poland; 4 Department of Psychiatry, Pomeranian Medical University in Szczecin, Szczecin, Poland

**Keywords:** Menopause, Quality of life, Personality, 5-HTT, *MAO*-*A*

## Abstract

**Background:**

Quality of life can be perceived as a subjective assessment of different aspects of human functioning. Personality is a factor which determines actions taken by individuals and their tendency to perceive reality in a particular way. Therefore, the assumption that personality may influence the QoL assessment seems reasonable. Our purpose was to assess the relationships between personality traits and the presence of the 44-bp VNTR polymorphism in the 5-HTT *(SLC 6A4)* promoter region and the 30-bp VNTR polymorphism in the *MAO*-*A* promoter region. We also wanted to determine the influence of personality on the quality of life of postmenopausal women.

**Methods:**

The study involved 214 postmenopausal women from northwest Poland. It was conducted using the NEO-FFI and the SF-36 questionnaires. DNA polymorphisms were identified using the polymerase chain reaction (PCR).

**Results:**

The average age of the women was 56.8 ± 4.08 years. Half of the respondents had completed second-level education, 69.2 % had life partners, and 53.3 % were professionally active. Women with the *3/3* genotype were characterized by significantly lower openness to experience than respondents with other *MAO*-*A* genotypes (*p* < 0.05). There was a significant correlation between the quality of life and the levels of neuroticism and extroversion, as well as between selected quality of life domains and the levels of agreeableness and conscientiousness.

**Conclusions:**

(1)Women with the *3/3* genotype of the 30-bp VNTR polymorphism in the *MAO*-*A* promoter region are characterized by lower levels of openness to experience than women with other *MAO*-*A* genotypes in our study (2) Personality traits may contribute to the assessment of the quality of life.

## Background

The postmenopausal period is a special time in a woman’s life—the time which entails progressive deterioration in the quality of life, leading as a consequence to lower physical activity, withdrawal from social life and unwillingness to develop. When analyzing inconveniences of the perimenopausal period, special attention should be paid to the fact that as many as 90 % of women suffer from mental disorders manifested by sudden mood changes, difficulties in coping with stress in everyday life, depressed mood, getting tired quickly, restlessness and irritation, as well as concentration problems, the weakening of memory, treatment unresponsive somatic complaints, and full-blown depression [1, 2]. The mechanism of developing mental disorders in the perimenopausal period has not been so far fully elucidated. It has not been unambiguously established whether lower mood, tension, and concentration problems are associated with changes in hormone metabolism, or whether they result from the configuration of psychosocial factors. A decline in the estrogen level results in vasomotor symptoms—such as hot flushes, sweats, and headache—which negatively affect the quality of women’s lives, causing sleep and concentration problems, oversensitivity, fatigue, and melancholy [3]. According to some reports, depression occurs mainly in those perimenopausal women who are predisposed to it and who have already experienced depressive episodes. The level of estrogen may contribute to depressive symptoms in the early stages of menopause, which suggests that a sudden drop in the estrogen level increases a risk of depressive disorders. It would explain the lack of relationship between depression and gradually decreasing estrogen levels in postmenopausal women. Depression in the postmenopausal period seems to have different aetiology [4, 5]. It is believed that the monoamine imbalance, which modifies the expression of traits, mental functions and behaviour, may contribute to mood disorders [6]. Scientists identified genes that may have the most profound effects on the metabolism of monoamine neurotransmitters, such as serotonin (5-HT) and monoamine oxidase A (MAO-A). The expression of these genes depends on the type of polymorphism potentially contributing to the occurrence of specific mood disorders. The serotonin transporter (5-HTT) gene (*SLC 6A4*) polymorphism is characterized by the insertion or deletion of a 44-bp fragment, and thus, the creation of the long (l) allele and the short (s) allele. The presence of the ‘s’ allele of the 5-HTT gene is presumably associated with a lower gene expression and worse functioning of the serotonin transporter, which in turn disturbs neurotransmission and results in a higher tendency to anxiety and avoidant behaviours [7, 8]. It has also been proved that the ‘s’ allele involves lower serotonergic flexibility and as a consequence impulsive behaviours [9]. MAO-A is an enzyme involved in the degradation of biogenic amines. Until now, researchers have mainly focused on the functional VNTR polymorphism in the promoter region of the gene encoding this enzyme. It consists of a 30-bp repeated sequence that may be present in 3, 3.5, 4, or 5 copies. The ‘3’ allele is associated with a lower, and the ‘3.5’, ‘4’, and ‘5’ alleles—with a higher gene transcriptional activity. The *MAO*-*A* gene may be responsible for an inclination to depression [10].

Based on analysis of the correlations between personality traits of monozygotic and dizygotic twins, and the phenomenon of sharing personality traits with brothers and sisters in biological and adoptive families, behavioural geneticists proved that the occurrence of similar personality traits within one family is determined by genetic factors. Personality consists of several dimensions or characteristics, which have a normal distribution in the general population. Personality is defined as an individual’s unique behavioural pattern. There are significant interindividual differences in reacting to changes in the external and internal environment. According to McCrae and Costa there are five personality dimensions: Neuroticism, Extroversion, Openness to experience, Agreeableness, and Conscientiousness [11].

At present, quality of life is the most dynamically developing and increasingly described measure of health. It defines the level of self-realization and satisfaction with life from a holistic perspective [12]. In the case of perimenopausal women, several aspects of QoL can be discussed, namely the subjective perception of one’s position in life, health status, as well as physiological changes and their consequences [13–15]. Somatic and psychological complaints usually change people’s views of their QoL. Currently, a lot of attention is devoted to the concept of health-related quality of life (HRQoL), which covers four domains: physical functioning, mental functioning, social functioning, and symptoms associated with the pathological and therapeutic processes. Self-reported QoL may change with time and under the influence of objective factors [16, 17]. Personality significantly determines the QoL assessment both among healthy patients, and those with somatic and/or mental disorders [18].

The purpose of this study was to determine how personality traits of postmenopausal women are related to the presence of the 44-bp VNTR polymorphism in the 5-HTT *(SLC 6A4)* promoter region and the 30-bp VNTR polymorphism in the *MAO*-*A* promoter region. We wanted also to establish the influence of personality on self-reported quality of life.

## Methods

Our research involved 214 healthy women, living in northwest Poland. All participants gave informed consent to take part in the study and their anonymity was preserved. The inclusion criteria were at least 1 year after the last menstruation, no alcohol abuse, no smoking, no endocrine disorders, no neoplastic diseases, and no current or past history of psychiatric treatment. To exclude mental disorders in the study group, all women were screened by means of the Primary Care Evaluation of Mental Disorders Patient Heath Questionnaire 9 (PRIME-MD PHQ-9) prior to the study. The PRIME-MD questionnaire concerns all criteria for depression diagnosis, and includes a gradual scale for measuring the severity of symptoms.

The first stage of the study was based on a survey performed using standard research instruments, namely the Neuroticism-Extroversion-Openness-Five Factor Inventory (NEO-FFI) and the Short Form Health Survey (SF-36) for measuring quality of life. The NEO-FFI is applied to analyze personality traits included in the Five Factor Model. The questionnaire consists of five scales measuring: neuroticism, extroversion, openness to experience, agreeableness and conscientiousness. Sixty self-descriptive statements are answered on a five-point scale. The points obtained for each of the NEO-FFI scales are summed up, thus giving the score: high (7–10), average (4–6), or low (1–3) for each of the five scales. The questionnaire was adapted into Polish by Bogdan Zawadzki, Jan Strelau, Piotr Szczepaniak, and Magdalena Śliwińska in 1998. The Short Form Health Survey (SF-36) serves for measuring quality of life. It consists of 11 questions, including 36 statements, divided into subscales measuring eight aspects of QoL, namely physical functioning, role physical, bodily pain, general health, vitality, social functioning, role emotional, and mental health.

The second stage of the study was based on genetic tests. For genetic analysis 10 ml venous blood samples were collected with the Vacutainer. Biological material (blood) was collected and stored in accordance with the principles of the quality management system of the Genetic Laboratory, the Department of Psychiatry. DNA was isolated from whole blood by the salting-out method of Miller. Polymerase chain reaction (PCR) was used to identify DNA polymorphisms. The aim of the analysis was to amplify the fragment consisting of 2–5 repetitions of the 30-bp VNTR polymorphism in the *MAO*-*A* promoter region. The following primer sequences were used: *MAO*-*A F: 5′ CCC AGG CTG CTC CAG AAA 3′*, and *MAO*-*A R: 5′ GGA CCT GGG CAG TTG TGC 3′*. The sizes of the amplified fragments were as follows: 239, 209, 226, and 269 bp. In the 5HTT polymorphism analysis, the fragment—including the 44-bp ins/del in the regulatory sequence (the presence or lack of 44-bp)—was amplified. The following primer sequences were used: *HTT F: 5′ GGC GTT GCC GCT CTG AAT GC 3′*, and *HTT R: 5′ GAG GGA CTG AGC TGG ACA ACC AC 3′*. The sizes of the amplified fragments were 484 and 528 bp.

Statistical analysis was performed using Statistica 7.1 PL. The following tests were applied: the Chi square independence test, the Kruskal–Wallis test, Pearson’s linear correlation coefficient, and the multiple comparison test, showing which groups differ from each other if the Kruskal–Wallis test demonstrated significant differences between at least two groups. The significance level was set at *α* = 0.05. The gene polymorphisms did not show significant deviations from Hardy–Weinberg’s Equilibrium. The study described in the article has been carried out in accordance with The Code of Ethics of the World Medical Association (Declaration of Helsinki) and was approved by the Bioethical Commission of the Pomeranian Medical University in Szczecin, Poland (Permission Number KB-0012/155/13).

## Results

The mean age of the women was 56.8 ± 4.08 years. Nearly half of them (47.7 %) had secondary education, 36.4 %—higher education. The majority of the respondents lived in cities with a population of more than 100,000 residents (61.7 %). Most women in the study (69.2 %) had life partners. Greater than half of the respondents (53.3 %) were professionally active. Low and medium levels of neuroticism were noted in 77.9 % of the women, and high levels in 22.1 %. About half of the women had medium levels and about 40 %—high levels of extroversion, agreeableness and conscientiousness. Medium levels of openness to experience were observed in 41.5 % of the respondents and high levels in 46.9 %.

Furthermore, we evaluated quality of life using the SF-36 questionnaire. The highest scores were obtained on physical functioning (79.07 ± 19.43), emotional functioning (74.84 ± 38.85), and social functioning (74.64 ± 23.31). The lowest scores were obtained on the vitality domain (57.26 ± 15.19), role physical (57.32 ± 42.92), and on general health (59.57 ± 18.66).

The results were statistically analysed with regard to the genotype distribution of the 44-bp VNTR polymorphism in the 5-HTT *(SLC 6A4)* promoter region and the expression of personality traits according to the NEO-FFI. The analysis conducted using the Kruskal–Wallis test did not reveal any statistically significant differences (Table 1).

**Table 1:** Kruskal–Wallis test results for neuroticism, extroversion, openness to experience, agreeableness, conscientiousness and the genotypes analysed

NEO-FFI	Genotypes			H (2, *n* = 208)	*p*	Genotypes			H (2, *n* = 208)	*p*
	$\bar{\chi }$ ± SD					$\bar{\chi }$ ± SD				
	l/s (*n* = 91)	s/s (*n* = 30)	l/l (*n* = 87)			3/3 (*n* = 28)	3/4 (*n* = 90)	4/4 (*n* = 90)		
Neuroticism	19.8 ± 7.7	19.9 ± 6.5	20.5 ± 8.3	0.42	>0.05	19.6 ± 6.8	20.6 ± 7.8	19.8 ± 8.0	0.59	>0.05
Extroversion	27.6 ± 6.4	26.9 ± 6.0	26.9 ± 6.7	0.43	>0.05	28.5 ± 6.6	26.9 ± 6.8	27.1 ± 6.1	0.98	>0.05
Openness to experience	27.6 ± 5.9	26.6 ± 6.9	26.7 ± 5.8	1.62	>0.05	24.3 ± 5.1	27.6 ± 5.8	27.5 ± 6.3	7.13	<0.05
Agreeableness	33.0 ± 4.6	33.0 ± 4.1	32.7 ± 4.5	0.45	>0.05	33.4 ± 4.6	33.1 ± 4.7	32.5 ± 4.2	1.59	>0.05
Conscientiousness	34.5 ± 6.1	34.5 ± 6.5	33.1 ± 6.1	2.09	>0.05	35.1 ± 6.5	33.5 ± 6.2	33.5 ± 6.2	1.96	>0.05

*H* Kruskal–Wallis test statistics, *p* level of significance for H

Similarly, the statistical analysis conducted using the Kruskal–Wallis test with regard to the genotype distribution of the 30-bp VNTR polymorphism in the *MAO*-*A* promoter region and the levels of personality traits according to the NEO-FFI demonstrated that the genotype distribution of the 30-bp VNTR polymorphism in the *MAO*-*A* promoter region had significant effects on the level of openness to experience (*p* < 0.05) (Table 1). Further analysis based on the test of multiple comparisons showed that women with the *3/3* genotype were characterized by significantly lower levels of openness to experience than respondents with other *MAO*-*A* genotypes (*p* < 0.05) (Table  2).

**Table 2:** Multiple comparison test results for openness to experience and *MAO*-*A* genotypes

*p* Value for multiple comparisons			
*MAO*-*A*	3/3	3/4	4/4
3/3		0.04	0.03
3/4	0.04		1.00
4/4	0.03	1.00	

*p* level of significance

We analyzed the influence of personality traits on the QoL of the surveyed, and found that the levels of neuroticism and extroversion positively correlated with all SF-36 QoL domains. We also observed a positive correlation between the level of agreeableness and all SF-36 QoL domains, except for the bodily pain domain, and positive correlations between the level of conscientiousness and the domains of physical functioning, vitality, social functioning, role emotional, and mental health. There was no statistically significant correlation between the women’s QoL and the openness to experience level (Table 3).

**Table 3:** Correlations between quality of life and personality traits according to the NEO-FFI

Domain	NEO-FFI									
	Neuroticism		Extroversion		Openness to experience		Agreeableness		Conscientiousness	
	*r*	*p*	*r*	*p*	*r*	*p*	*r*	*p*	*r*	*p*
Physical functioning	−0.26	<0.05	0.19	<0.05	−0.01	> 0.05	0.18	<0.05	0.11	>0.05
Role physical	−0.30	<0.05	0.29	<0.05	0.03	> 0.05	0.18	<0.05	0.19	<0.05
Bodily pain	−0.36	<0.05	0.23	<0.05	0.05	> 0.05	0.12	>0.05	0.00	>0.05
General health	−0.41	<0.05	0.33	<0.05	0.07	> 0.05	0.14	<0.05	0.08	>0.05
Vitality	−0.51	<0.05	0.54	<0.05	0.06	> 0.05	0.20	<0.05	0.26	<0.05
Social functioning	−0.52	<0.05	0.39	<0.05	0.11	> 0.05	0.27	<0.05	0.18	<0.05
Role emotional	−0.53	<0.05	0.41	<0.05	0.06	> 0.05	0.19	<0.05	0.24	<0.05
Mental health	−0.66	<0.05	0.51	<0.05	0.11	> 0.05	0.21	<0.05	0.27	<0.05

*r* Pearson’s linear correlation coefficient, *p* level of significance for r statistics, *n* number of participants

## Discussion

Numerous studies conducted so far confirm the influence of genetic factors on personality. Many of them describe the role that the *MAO*-*A* gene polymorphisms play in the evolution of personality traits. The analysis of 277 women and men, performed by Chester et al., demonstrated that the presence of the low activity *MAO*-*A* predisposes to aggressive behaviour and acting on negative impulses [19]. According to Caspi et al., individuals with the low-activity *MAO*-*A* genotype who were hurt and maltreated in their childhood significantly more often develop conduct disorders and display antisocial behaviour than their counterparts with the high-activity *MAO*-*A* genotype [20]. These findings were supported by Foley et al. and Nilsson et al. [21, 22]. Other researchers assert that people with the low-activity *MAO*-*A* and the *5*-*HTTLPR* l/l genotype are less impulsive than those with the low-activity *MAO*-*A* and the s/s genotype [23]. It was also found that men with the low activity *MAO*-*A* gene have higher levels of neuroticism [24], and women with the high activity *MAO*-*A* have higher levels of anxiety and suffer from more severe depression [25, 26]. Additionally, there are reports that the *MAO*-*A* genotype contributes to the occurrence of antisocial behaviour among female teenagers with alcohol problems [27]. Our study did not provide evidence for the impact of the investigated gene polymorphisms on the levels of personality traits. The only exception was the 3/3 genotype of the 30-bp VNTR polymorphism in the *MAO*-*A* promoter region, whose presence contributed to a higher openness to experience level.

The presence of the ‘s’ allele of the 5-HTT gene may be associated with a higher tendency to anxiety, as well as avoidant and impulsive behaviour [7–9]. The study based on magnetic resonance imaging (MRI) demonstrated that carriers of one or two low transcriptional activity alleles (the s/s or s/l genotype) had higher neuronal activity of the amygdala responsible for negative emotions, aggression, and defence reactions in response to stressful life events, than their counterparts with a higher transcriptional activity allele (the l/l genotype) [28]. Some researchers claim that girls carrying the ‘s’ allele and getting no support from their families more often behave without inhibitions and engage in impetuous and ill-considered actions. At the same time, family relationships were proved to have no influence on the occurrence of impulsive behaviours in girls with the ‘l’ allele [29]. Our study did not reveal any statistically significant differences in the genotype distribution of the 44-bp VNTR polymorphism in the 5-HTT *(SLC 6A4)* promoter region depending on the levels of personality traits as gauged by the NEO-FFI. Similar results were obtained by Stein et al., who searched for the connection between the *5*-*HTTLPR* gene polymorphism and the level of neuroticism (a statistically significant relationship was not demonstrated) [7]. Gingnell et al. reported that high neuroticism was more common among women suffering from premenstrual dysphoric disorder (PMDD) than in the group of healthy females. What is more, anxiety and the lack of assertiveness were more common among women with the ‘s’ allele than those with the ‘l’ allele [30]. Jacob et al. found that the levels of neuroticism were higher in a group of patients with type C personality disorders who carried the ‘s’ allele than in those without this allele. The study did not reveal differences in the distribution of the 5-HTT genotypes between respondents with types A and B personality disorders and those in the control group [31].

There is no doubt that the quality of women’s lives deteriorates in the perimenopausal period. The contribution of climacteric symptoms to a decline in the quality of life (especially psychosocial, physical and sexual spheres) has been confirmed by many studies [32, 33]. Little attention has been so far paid to personality, which undoubtedly plays a role in the QoL assessment. Van Straten et al. used the SF-36 questionnaire to measure the QoL of 640 outpatients with mood and anxiety disorders with regard to personality factors. They found that all personality traits according to the NEO-FFI, except for conscientiousness, were closely related to the QoL. The level of agreeableness was related to the assessment of physical and social domains, while the levels of neuroticism, extroversion, and openness to experience were related to mental domain. The low QoL level was not only associated with mood and anxiety disorders but also these personality traits which are relatively stable throughout life [34]. In the study of 442 primary care patients, aged 65 years or more, conducted using the NEO-FFI and the SF-36, high levels of neuroticism were accompanied by lower QoL in the physical sphere. High levels of conscientiousness, on the other hand, were associated with higher SF-36 scores [35]. The study of over 5000 adults, aged 50 years, carried out by Cheng et al. revealed a significant relationship between personality traits and the QoL assessment [36]. Extroversion and conscientiousness were significantly related to the QoL in the study group. Besiland et al. analysed factors contributing to the QoL of 260 patients after surgical treatment for kidney cancer. In this analysis, the QoL was the lowest assessed by patients with neurotic personality and avoidant attitudes [37].

In our study, neuroticism and extroversion affected all QoL domains, agreeableness influenced all domains except for bodily pain, and conscientiousness had effects on all domains except for physical functioning, general health and vitality. There was no relationship between QoL and openness to experience.

Quality of life can be perceived as a subjective assessment of different aspects of human functioning. Personality is a factor which determines actions taken by individuals and their tendency to perceive reality in a particular way. Therefore, the assumption that personality may influence the QoL assessment seems reasonable. Our findings confirm that personality traits have effects on various QoL aspects. Based on our results and those reported by other authors, it can be asserted that QoL does not depend exclusively on health status and life situation, but also personality traits which are relatively stable throughout life.

## Conclusions

1. Women with the 3/3 genotype of the 30-bp VNTR polymorphism in the *MAO*-*A* promoter region are characterized by lower levels of openness to experience than women with other *MAO*-*A* genotypes in our study.
2. Personality traits may contribute to the assessment of the quality of life.

Our findings suggest that either monoamine imbalance—associated with the presence of the 30-bp VNTR polymorphism in the *MAO*-*A* promoter region and the 44-bp VNTR polymorphism in the 5-HTT *(SLC 6A4)* promoter region—has no influence on the QoL of postmenopausal women, or the relationships between these polymorphisms, QoL, and personality traits were not observed because the study involved only healthy women (those with mental disorders were excluded).

## Limitations

The size of the study sample does not let us draw the conclusions for the whole population.

Another limitation of the study is the lack of personality assessment according to the Structured Clinical Interview for DSM-IV Disorders (SCID), which would allow us to make the major DSM-IV Axis I diagnosis.

## Abbreviations

5-HTT: serotonin transporter
COPE: a multidimensional coping inventory to assess the different ways in which people respond to stress
EORTC-QLQ-C30: European organization for research and treatment of cancer quality of life questionnaire
MAO-A: monoamine oxidase A
MHT: menopausal hormone therapy
NEO-FFI: neuroticism-extraversion-openness five-factor inventory
PCR: polymerase chain reaction
PRIME-MD: primary care evaluation of mental disorders patient health questionnaire 9
SF-36: short form health survey
SLC6A4: solute carrier family 6 member 4
TCI: temperament and character inventory
VNTR: variable number of tandem repeats
WHOQoL-BREF: World Health Organization quality of life-bref
